# Novel herpesvirus potentially associated with oral lesions in a two-toed sloth (*Choloepus didactylus*)

**DOI:** 10.1007/s11259-025-10983-7

**Published:** 2025-11-26

**Authors:** Carlos Sacristán, Laura Reisfeld, Aricia Duarte-Benvenuto, Roberta Zamana-Ramblas, Paloma Canedo, Islene Silva Santos, Jordana Barros, Fabiana Padilha, José Luiz Catão-Dias, Ana Carolina Ewbank

**Affiliations:** 1 Animal Health Research Center, INIA-CSIC, Valdeolmos, Madrid, Spain; 2 Aquario de São Paulo, São Paulo, Brazil; 3 School of Veterinary Medicine and Animal Science, University of São Paulo, São Paulo, São Paulo, Brazil

**Keywords:** *Candida* spp., Mycosis, Gammaherpesvirus, Sloth, South America, Xenarthra

## Abstract

The superorder Xenarthra comprises anteaters, sloths and armadillos, all endemic of the Americas. Despite their distinctive evolutionary history, information regarding viruses affecting this group remains scarce. Herein, we report a gammaherpesvirus infection in a two-toed sloth (*Choloepus didactylus*) under human care that presented recurrent oral vesicular lesions and oral candidiasis. The gammaherpesvirus identified in oral swab sample likely represents a novel species, due to the large genetic divergence when compared to the closest sequences available on public databases, and its identification in a novel host species. This finding broadens the herpesvirus host range in Xenarthra, representing the third description in this taxonomic group. Future studies should elucidate the pathogenicity of this novel herpesvirus.

## Background

Herpesvirus (order *Herpesvirales*) are large linear double-stranded DNA viruses able to establish latency in their natural host species (Davison, 2010). *Orthoherpesviridae*, one of the main herpesvirus families, comprise three subfamilies - *Alphaherpesvirinae*, *Betaherpesvirinae* and *Gammaherpesvirinae*, and infects reptiles, birds and mammals (Davison, 2010). Herpesviruses typically co-diverge with their host species; however, host shifts can also occur (Kaján et al., 2020; Brito et al., 2021). In immunocompetent natural hosts, herpesviruses generally cause mild lesions or subclinical infections (Kaleta & Docherty, 2007; Sacristán et al., 2024). Nevertheless, in non-adapted species or immunocompromised individuals, these viruses can cause severe and even fatal disease (Kaleta & Docherty, 2007; Sacristán et al., 2024).

To date, herpesviruses have been described in the mammalian subclass Theria, i.e., in the infraclass Marsupialia and Placentalia, the latter including the superorders Laurasiatheria (e.g., dolphins), Afrotheria (e.g., elephants, West Indian manatees *Trichechus manatus*), Euarchontoglires (e.g., gorillas) and Xenarthra (Ehlers et al., 2008; Sacristán et al., 2019; Vaz et al., 2019; Navas-Ruárez et al., 2022; Ewbank et al., 2023). There are no reports of herpesviruses in the order Monotremata. Regarding xenarthrans, a group composed of anteaters, sloths and armadillos, there is currently one herpesvirus report - in giant armadillos (*Priodontes maximus*) (Navas-Suárez et al., 2022). Additionally, although these sequences are not available on public databases, ten reads were assigned to *Orthoherpesviridae* family using Kraken2 in a metagenomics study performed with pooled fecal samples of southeastern maned sloths (*Bradypus crinitus*) (Coimbra et al., 2025). Herein, we describe a herpesvirus infection in a two-toed sloth (*Choloepus didactylus*) under human care.

## Case presentation

An orphaned male two-toed sloth of approximately two months of age was found near the Tarumã River, in Manaus, Amazonas state, Brazil, on July 14th, 2020. The animal was taken to a rehabilitation center for care, and a retrognathic condition was diagnosed. In November 2021, the animal was transferred to the Aquário de São Paulo, São Paulo, Brazil, where it was individually housed.

**Fig. 1:**
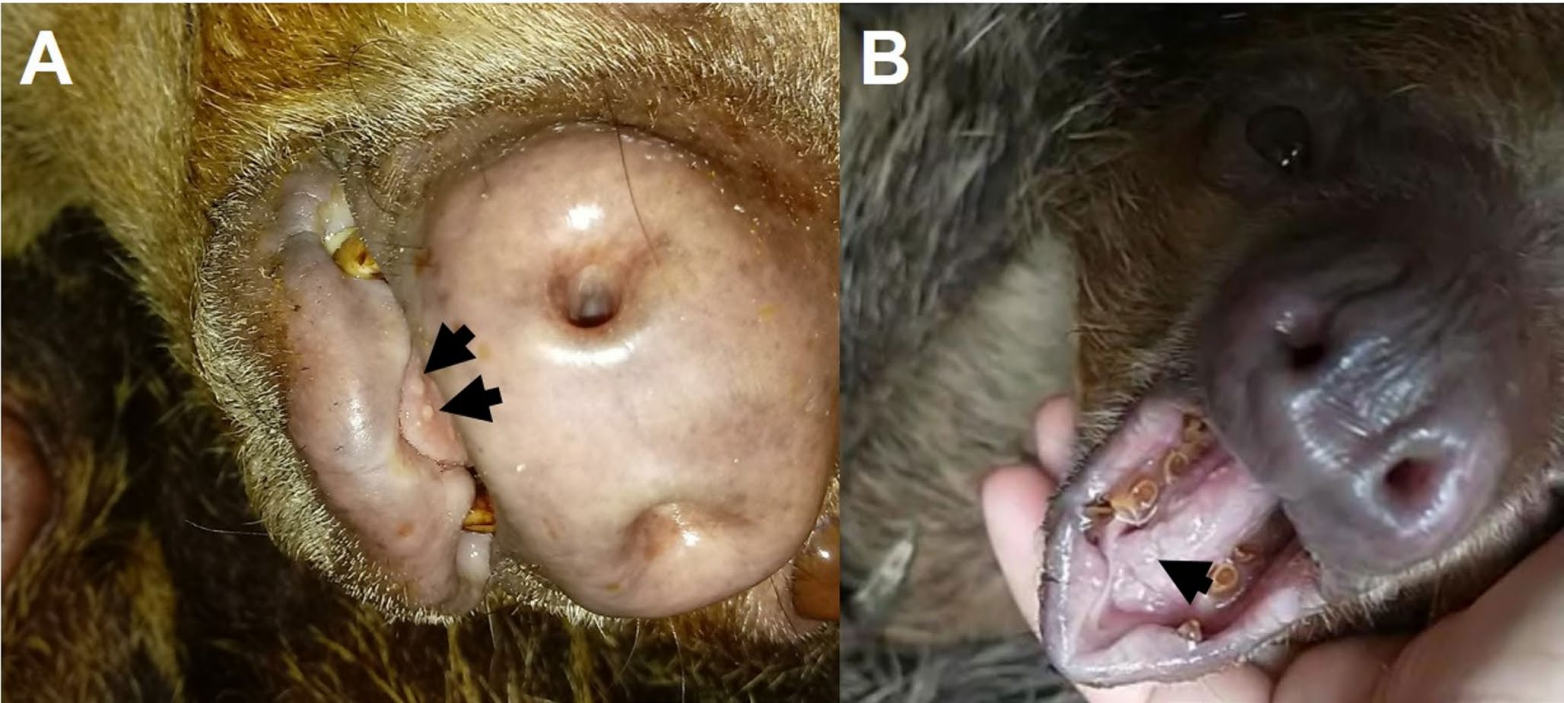
(**A**, **B**) Vesicular lesions on the tongue of a two-toed sloth (*Choloepus didactylus*), marked with black arrows

On October 18th, 2022, the animal presented pruritus on the palate and tongue, and multifocal whitish plaques were observed on its tongue (Fig. 1). Oral cavity swab samples were taken for cytology, and bacterial and fungal culture. Cytology revealed the presence of budding yeast-like structures (2–4 per field) with rare pseudohyphae, confirmed as *Candida* sp. by culture on micobiotic agar and Sabouraud agar. Additionally, cytological analysis demonstrated gram-negative cocci and bacilli (65%), and gram-positive bacilli (30%) and cocci (5%). Bacterial culture further identified *Providencia alcalifaciens* and *Corynebacterium* spp. The lesions healed after a 7-day treatment with oral nystatin (Teuto, Anápolis, Brazil, 20.000 UI/kg, bid) and blue LED laser therapy (1-minute sessions three times a week, whenever the individual presented oral cavity lesions). *Echinacea* 6CH (4 globules BID for 30 days), and *Chamomilla* 6CH (4 globules BID for 15 days) were administered as part of the supportive treatment.

The animal suffered another episode in May 23th, 2023, presenting decreased appetite. Cytology revealed the presence of pseudohyphae, and the animal was treated with the protocol described above, as well as with blue LED laser therapy, and oral dipyrone (Laboratório EMS, Hortolândia, Brazil) 25 mg/kg SID whenever signs of discomfort were present.

In November 25th, 2023, the animal presented low food intake, and discrete swelling of the lower lip, whitish diffuse spots on the palate and whitish plaques on the tongue. Treatment comprised a single dose (0.1 mg/kg IM) of meloxicam (Ourofino, São Paulo, Brazil) administered orally at 0.1 mg/kg SID and blue LED laser therapy applied to the oral cavity. Cytology and fungal culture of an oral swab were negative; however, bacterial culture isolated *Klebsiella* spp. predominantly accompanied by normal microbiota. The animal did not present biochemistry or leukocyte differential count changes. An oral swab sample was collected for herpesvirus PCR on the 31 st of that month.

On December 6th, 2023, the animal presented multifocal round lesions of approximately 0.5 cm in diameter on the tongue and palate (aphthae), with signs of discomfort and low appetite. The individual received meloxicam 0.1 mg/kg IM as a single dose, fluid extract of chamomile Admuc (Avert-Biolab, São Paulo, Brazil) applied to the oral lesions, and blue LED laser therapy. Based on the gross appearance of the lingual lesions, a sample was collected for herpesvirus PCR. There were no apparent housing or environmental changes after the arrival of the animal at the Aquário de São Paulo.

Briefly, DNA of an oral swab was extracted using the DNeasy Blood and Tissue kit (Qiagen, Hilden, Germany), according to the manufacturer’s instructions. Subsequently, a broad-range herpesvirus PCR was performed to partially amplify a 230–330 fragment of the DNA polymerase gene of the subfamilies *Alphaherpesvirinae*, *Betaherpesvirinae* and *Gammaherpesvirinae* (VanDevanter et al., 1996). Amplicons of the expected size were purified using ExoSAP-IT (Applied Biosystems, Foster City, CA, USA) and directly sequenced in both directions. The obtained forward and reverse sequences were aligned using MEGA11 in order to construct the consensus sequence, which was compared to those available at public databases using BLAST search. After excluding primer sequences, a maximum likelihood amino acid tree was constructed using the sequence identified in this study, other herpesvirus from xenarthrans, and species of the subfamilies *Alphaherpesvirinae*, *Betaherpesvirinae* and *Gammaherpesvirinae* recognized by the International Committee on Taxonomy of Viruses.

**Fig. 2:**
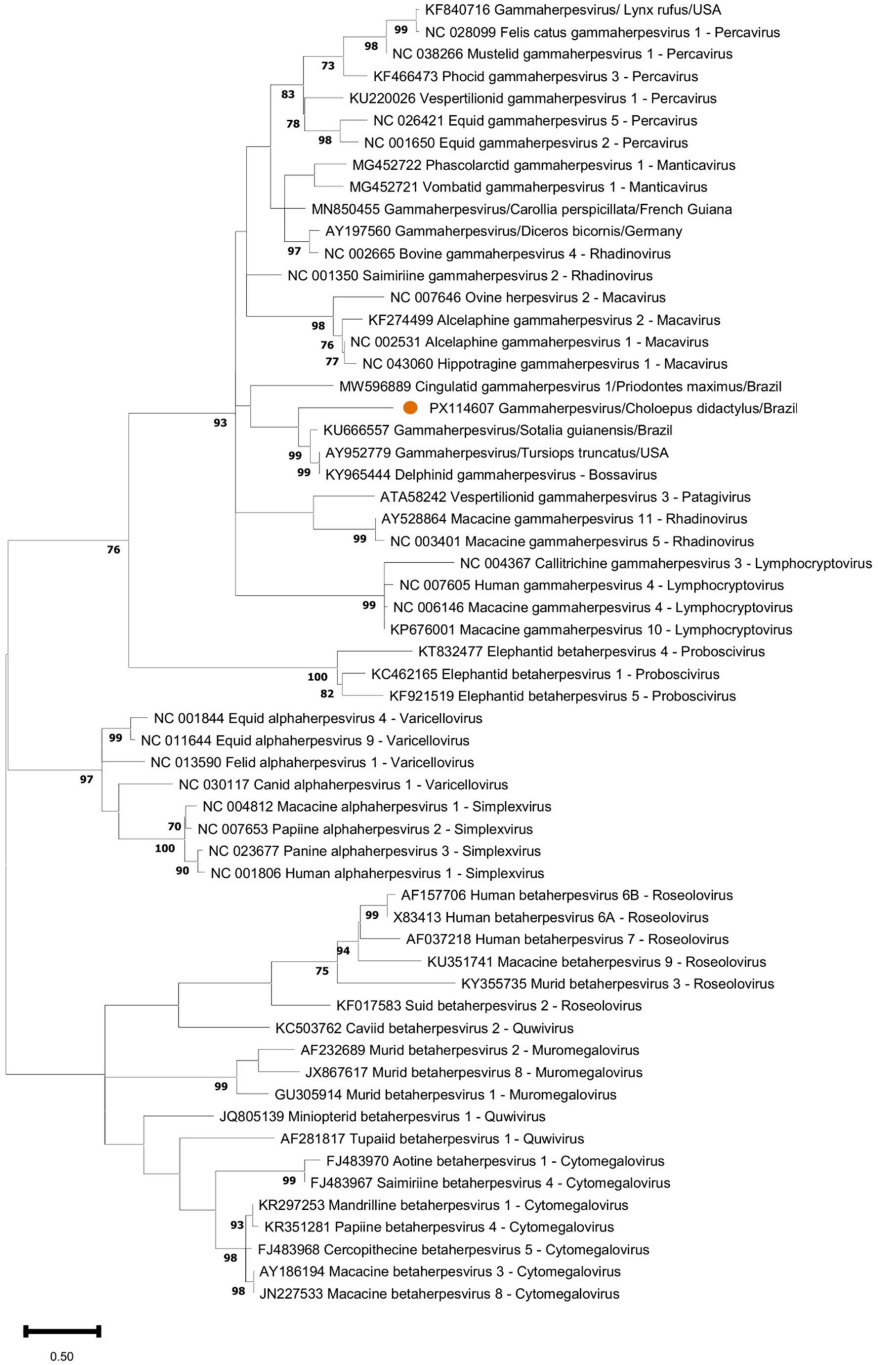
Maximum likelihood phylogenetic tree of the amino acid alignment of: (i) the herpesvirus sequence obtained from a two-toed sloth (*Choloepus didactylus*) in this study (orange dot), (ii) the closest sequences available at the GenBank/ENA/DDBJ databases (AY197560, MN850455), (iii) the only known herpesvirus sequence available from a xenarthran (MW596889), and (iv) representative alpha-, beta-, and gammaherpesvirus species recognized by the International Committee on Taxonomy of Viruses, indicating the viral genera. The evolutionary model employed was Le Gascuel with invariant sites and gamma distribution, selected using ProtTest. The tree was generated with 1,000 bootstrap replicates. Bootstrap values below 70 were omitted

A 157 bp sequence was identified in the oral swab of the two-toed sloth and submitted to GenBank under accession number PX114607. The sequence presented the highest nucleotide similarity (59.9%) to gammaherpesvirus sequences (AF287948, AY197560) obtained in a captive black rhinoceros (*Diceros bicornis*) of USA and Germany, while the highest amino acid similarity (68.6%) was with a gammaherpesvirus identified in a Seba’s short-tailed bat (*Carollia perspicillata*, QJX19393, corresponding to the nucleotide sequence MN850455). The nucleotide and amino acid sequence similarities were 55.4% and 55.8%, respectively, when compared to the only known herpesvirus sequence available from a xenarthran (accession number MW596889), retrieved from a giant armadillo. On the phylogenetic tree, the two-toed sloth herpesvirus sequence was classified as a gammaherpesvirus, but did not cluster within any of the known gammaherpesvirus genera (Fig. 2).

### Discussion and conclusions

To the authors’ knowledge, this is the first report of a herpesvirus - specifically a gammaherpesvirus - in a two-toed sloth, and the third in xenarthrans worldwide. Due to the large genetic divergence when compared to the closest sequences available at public databases, and its identification in a novel host species, the gammaherpesvirus identified herein is likely a novel herpesvirus species, tentatively named two-toed sloth gammaherpesvirus. Although the initial lesions described in the animal were likely due to candidiasis, the vesicular lesions observed in subsequent episodes could be due to herpesviruses. Vesicular lesions are often associated with alphaherpesviruses (e.g., *Simplexvirus humanalpha1*, *Varicellovirus humanalpha3*) (Mehrmal et al., 2024); however, some gammaherpesviruses have also been detected from vesicular lesions, such as in horses - associated with equine gammaherpesvirus 5 (Peters-Kennedy et al., 2023), and those described by Lee et al. (2024) in two false killer whales (*Pseudorca crassidens*) in the Republic of Korea. Alternatively, the detection of this gammaherpesvirus in an oral swab could have been due to the animal’s immunosuppression - as evidenced by the candidiasis, and the subsequent reactivation of the virus, given that several gammaherpesviruses are excreted in saliva (Newton et al., 2018).

In summary, this study broadens the host range of herpesviruses in xenarthrans. Future studies should elucidate the pathogenicity of this novel herpesvirus.

## Data Availability

All data generated or analyzed during this research are included in the manuscript. The nucleotide sequence obtained in this research was submitted to GenBank under accession number PX114607.
